# Prediction of leg-length discrepancy in pediatric femoral shaft fracture using bone SPECT/CT: A case report

**DOI:** 10.1097/MD.0000000000035860

**Published:** 2023-11-03

**Authors:** Sung Il Wang, Hwan-Jeong Jeong, Seok Tae Lim, Yeon-Hee Han

**Affiliations:** a Department of Orthopaedics Surgery, Jeonbuk National University Medical School, Research Institute for Endocrine Sciences and Research Institute of Clinical Medicine of Jeonbuk National University-Biomedical Research Institute of Jeonbuk National University Hospital, Jeonju, Jeonbuk, South Korea; b Department of Nuclear Medicine, Research Institute of Clinical Medicine of Jeonbuk National University-Biomedical Research Institute of Jeonbuk National University Hospital, Jounbuk National University Medical School and Hospital, Jeonju, Jeonbuk, South Korea.

**Keywords:** bone SPECT/CT, femur fracture, growth plate, leg-length discrepancy

## Abstract

**Rationale:**

Children’s bones are in the process of growing in both length and width. Therefore, evaluating whether fractures affect the growth plate or not is very crucial. However, even in cases of lower limb fractures where the growth plate remains unaffected, overgrowth or shortening of the affected limb are encountered.

**Patient concerns:**

An 11-year-old boy was admitted to the emergency department after a passenger car accident.

**Diagnoses:**

A comminuted fracture of the right femoral shaft was diagnosed by X-ray image.

**Interventions:**

Closed reduction and internal fixation were performed using intramedullary titanium elastic nails. Six months after the operation, bone union was achieved and the nails were removed.

**Outcomes:**

Nine months after nail removal, the right leg was unexpectedly noticed 5 mm shorter than the left one. On the initial and follow-up bone single-photon emission computed tomography/computed tomography images with a 9-month interval, radioactivity of growth plate in the right proximal femur was much lower than that of the left femur, suggesting a further increasing of leg-length discrepancy (LLD) in the future. As we expected, LLD had progressively increased up to 20 mm. Epiphysiodesis was finally decided for the left distal femur. Twenty-two months after the length equalization operation, LLD decreased to 5 mm.

**Lessons:**

This case emphasizes that quantitative analysis of growth plate activity using bone single-photon emission computed tomography/computed tomography could predict LLD and help us decide when and which limb should be operated on for pediatric patients with lower limb fractures.

## 1. Introduction

Since children’s bones are in the process of growing in length, evaluating whether fractures affect the growth plate or not is very crucial. However, even in cases of lower limb fractures where the growth plate remains unaffected, overgrowth or shortening of the affected limb are encountered. Overgrowth of a fractured limb is much more common than shortening in pediatric lower limb fractures. Therefore, superimposition of fragments and intentional shortening techniques are empirically used during operation.^[1]^ In most cases, overgrowth of the fractured limb compensates for the initial shortening and reaches a length similar to that on the unaffected side.^[1]^ In pediatric patients, one important point that we should not overlook, unlike in adult, is the possibility of unexpected overgrowth or shortening even after achieving complete bone union. Here, we report a rare case where leg-length discrepancy (LLD) progressively worsened due to shortening on the affected limb after bone union. Furthermore, we aim to share the utility of quantitative analysis using bone single-photon emission computed tomography/computed tomography (SPECT/CT) in predicting LLD in pediatric patients with lower limb fractures.

## 2. Case presentation

An 11-year-old boy complained of severe right thigh pain after a passenger car accident. Based on his symptom and trauma history, a right femoral fracture was the first clinical consideration. There was direct tenderness on the right thigh. Range of motion in the right hip and knee was limited because of right thigh pain. However, motor and sensory functions were intact. Pulses on the right femoral artery and dorsalis pedis artery were also normal.

A plain X-ray taken in the emergency department revealed a comminuted fracture of the right femoral shaft. Emergent closed reduction and internal fixation were conducted with intramedullary titanium elastic nails. Plain X-rays were taken every 3 months to evaluate bone union. Six months after the operation, a union was finally determined on plain X-ray and the intramedullary nails were removed. However, during a physical examination conducted 9 months after nail removal, an unexpected finding was noted. The length of the right leg, measured from the anterior superior iliac spine to the medial malleolus using a tape measure, was observed to be 5 mm shorter than the left leg. An orthotic insole for the right lower limb was prescribed.

**Figure 1.**
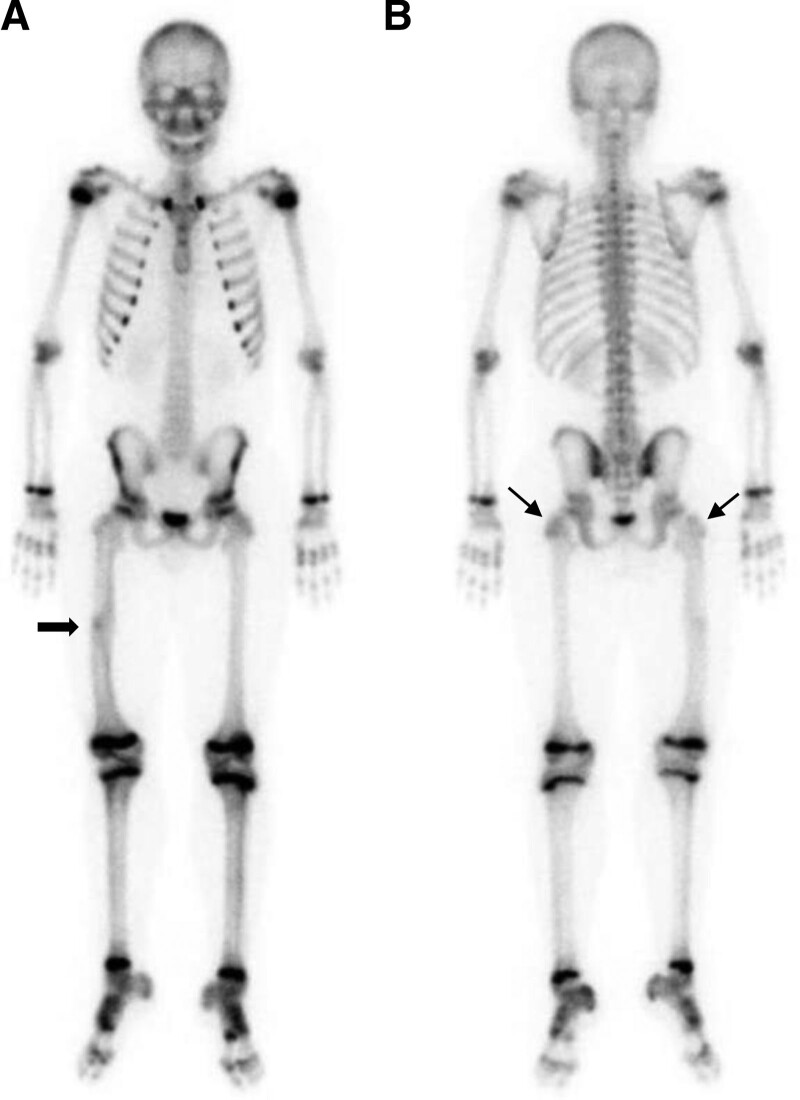
Whole-body bone scan at 9 months after nail removal. Focal increased uptake is observed at the previous fracture site in the right femoral mid shaft which is in a union state (A, thick arrow). There are no notable differences in the radioactivity of the growth plates in both femoral heads and distal femurs. However, on the posterior view, the radioactivity of the growth plate in the right greater trochanter appears lower compared to that of left femur (B, arrows).

To assess growth plate activity of the femurs, whole-body bone scan was performed using technetium-99m hydroxymethylene diphosphonate. Focal increased uptake was observed at the previous fracture site in the right femoral mid shaft which was in a union state (Fig. 1A, thick arrow). There were no notable differences in the radioactivity of the growth plates in both femoral heads and distal femurs. However, on the posterior view, the radioactivity of the growth plate in the right greater trochanter appeared lower compared to that of left femur (Fig. 1B, arrows).

**Figure 2.**
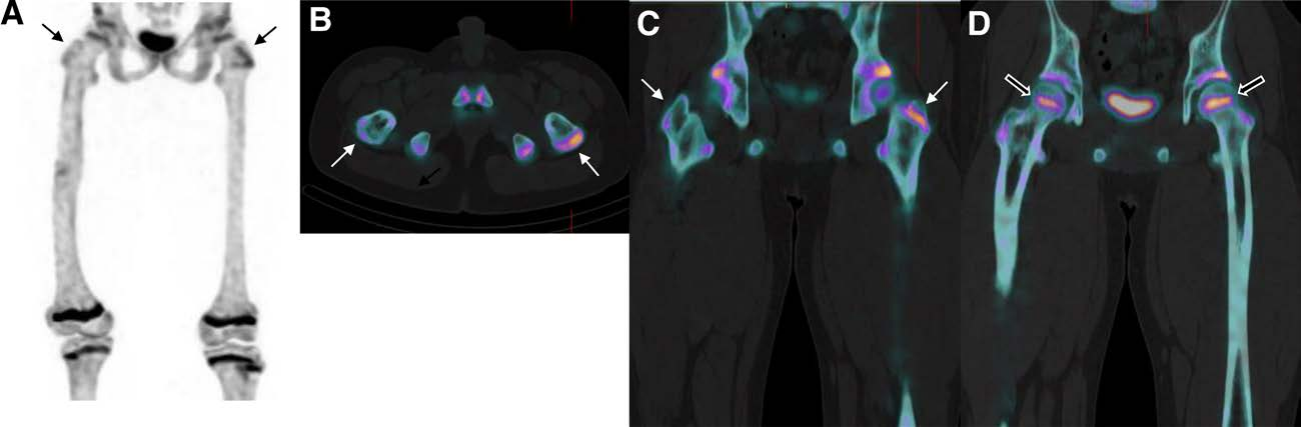
Initial bone SPECT/CT taken along with a whole-body bone scan. Radioactivities of the right greater trochanter and right femoral head were lower than those of the contralateral femur. Arrows show radioactivity in the growth plates of the greater trochanters, while empty arrows indicate those of the femoral heads. (A: maximal intensity projection (MIP) image; B and C: axial and coronal fusion images of the greater trochanters; D: coronal fusion image of the femoral heads).

Subsequent bone SPECT/CT demonstrated a clear reduction in the right greater trochanter (Fig. 2A–C). Furthermore, decreased radioactivity of the growth plate in the right femoral head, which was not clearly evident in the whole-body bone scan, was noticed (Fig. 2D). For quantitative analysis, ellipsoidal volumes of interest of the same size were placed on corresponding growth plates. Maximum standardized uptake value (SUVmax), mean standardized uptake value (SUVmean), and average radioactivity (Bq/mL) were obtained. All these radioactivities of growth plates in the right greater trochanter and right femoral head were lower than those of the left femur, suggesting a further increasing of LLD in the future (Table 1). On the other hand, in the radioactivity of the growth plate in the distal femur, SUVmean and average radioactivity showed similar values between the left and right sides, while SUVmax was higher on the right side.

**Table 1:** Radioactivity of growth plates.

Time after nail removal	Parameter	Greater trochanter		Femoral head		Distal femur	
		Right	Left	Right	Left	Right	Left
9 months	SUVmax	6.18	10.74	10.59	10.79	22.53	18.65
	SUVmean	2.85	3.93	4.9	5.38	5.81	5.65
	Average radioactivity (Bq/mL)	41,907.11	57,912.59	76,680.98	80,322.81	84,437.81	84,728.75
18 months	SUVmax	7.18	11.13	7.60	8.33	14.80	14.16
	SUVmean	3.08	4.51	4.49	4.89	5.01	4.74
	Average radioactivity (Bq/mL)	45,842.24	67,134.20	66,881.26	72,788.77	76,669.98	71,866.75

**Figure 3.**
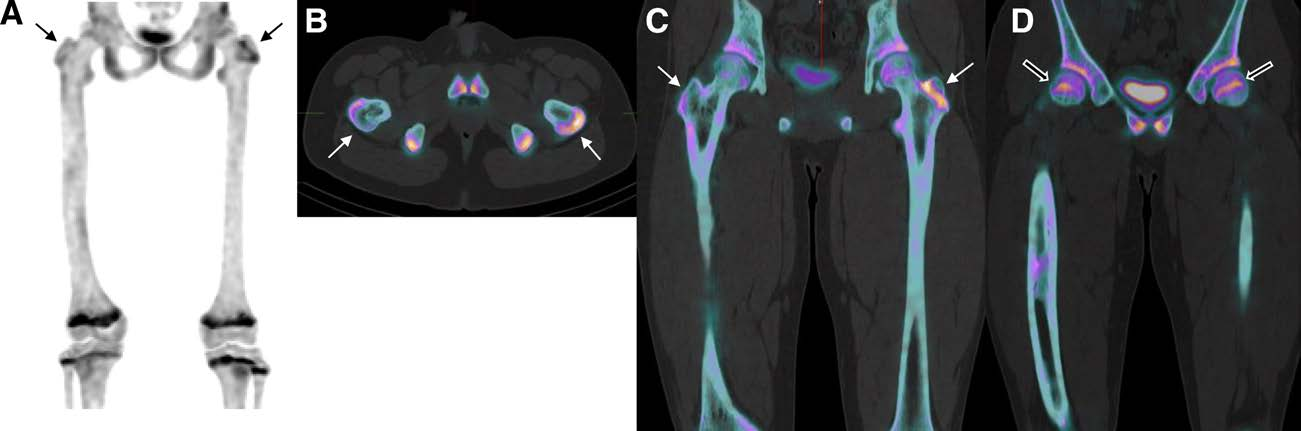
Follow-up bone SPECT/CT conducted 9 months after the initial bone SPECT/CT. Differences in the growth plate radioactivities between the proximal femurs became wider. Arrows show radioactivity in the growth plates of the greater trochanters, while empty arrows indicate those of the femoral heads (A: MIP image; B and C: axial and coronal fusion images of the greater trochanters; D: coronal fusion image of the femoral heads).

As expected, the LLD increased from the initial 5 mm to 20 mm during the 9-month follow-up period after the initial bone SPECT/CT. Before determining the treatment method, it was crucial to evaluate the femoral growth plate activity in the patient, so a follow-up bone SPECT/CT was performed. Radioactivities of growth plates in the right proximal femur remained lower compared to those of the left femur (Fig. 3 and Table 1). It was predictable that the LLD would continue to increase. A decision was made to perform a length equalization procedure named epiphysiodesis. Hemiepiphysiodesis plates were then carefully inserted on the medial and lateral areas of the left distal femoral epiphysis with the assistance of a C-arm intensifier. LLD gradually decreased from 20 mm to 15 mm within 5 months and from 15 mm to 5 mm over the next 10 months. The patient was instructed to use an orthotic insole of the appropriate thickness for the right leg. Hemiepiphysiodesis plates were finally removed 22 months after the epiphysiodesis procedure. No signs of scoliosis or abnormal gaits were observed during the 18 months following the plate removal.

## 3. Discussion

Femoral shaft fractures account for about 1.6% of all pediatric fractures.^[2]^ Treatment methods vary from simple casting to intramedullary nailing and plate fixation.^[3–7]^ In pediatric lower limb fractures, unlike in adults, evaluating lengthwise growth as well as a bone union is crucial because overgrowth or shortening of the affected limb is one of the critical complications. LLD owing to overgrowth or shortening over 15 mm could cause low back or pelvic pain, stress fractures, and scoliosis.^[8,9]^

In general, overgrowth of the fractured limb is more commonly observed than shortening. Age, fracture site, fracture stability, and nail-canal diameter ratio have been suggested as risk factors for femoral overgrowth.^[10]^ Many theories have been proposed to explain the reason for overgrowth, such as increased perfusion at the fracture site, nearby periosteum, and growth plates.^[11]^ Compared to overgrowth, shortening of the fractured limb is less common. It is known to appear after length-unstable fractures such as spiral, long oblique, and comminuted fractures, and is mainly explained by superimposition of the fracture lesion. However, in the present case, LLD was noticed and worsened after achieving complete bone union. Therefore, the superimposition of the fracture site could not be the reason for the shortening observed in our case.

In this case, during the radioactivities of the growth plates in the right proximal femur remained lower compared to those in the left femur, the LLD gradually increased by 15 mm over a 9-month period. Although the exact reasons for the reduced radioactivities in the fractured limb are unclear, it is suggested that excessive pressure at the time of trauma might have damaged the growth plates in the fractured limb.

Bone scan using technetium-99m diphosphonate radiopharmaceutical is the only imaging modality that can show growth plate activity. Efforts to assess growth plate activity through bone scans have been ongoing ever since its utilization.^[12]^ However, the interpretation of two-dimensional bone scan images heavily relies on the visual analysis by the interpreter, making it prone to overlooking subtle changes in growth plate activity. In addition, when evaluating fracture sites, hypertrophic nonunion can lead to a misconception of an appropriate ongoing union process due to the increased uptake of bone tracer.^[13]^

Since the advent of SPECT/CT, its clinical utility has been recognized across various fields and the musculoskeletal system utilizing bone SPECT/CT is no exception. Due to its high resolution, three-dimensional information, and anatomical details offered by bone SPECT/CT, it enables the detection of subtle differences in growth plate activity and allows accurate evaluation of the union process at the fracture site. Furthermore, recent advances in bone SPECT/CT provide the advantage of measuring various parameters including SUVmax, SUVmean, and peak standardized uptake value,^[14]^ which facilitate accurate quantitative analysis. In our case, bone SPECT/CT demonstrated its ability to identify additional finding that was missed on the whole-body bone scan, in addition to clearly revealing the ambiguous finding observed in the two-dimensional bone scan.

Quantitative analysis of growth plate activity using bone SPECT/CT can play a crucial role in determining treatment methods and predicting the prognosis of pediatric patients with lower limb fractures. Therefore, in cases where an unexpected course is noticed or ambiguous findings are observed in whole-body bone scans, considering bone SPECT/CT may be beneficial. Additionally, strict adherence to pediatric bone SPECT/CT imaging guidelines is essential to ensure radiation protection.

In conclusion, this case highlights that quantitative analysis of growth plate activity using bone SPECT/CT could predict LLD and help us decide when and which limb should be operated on in pediatric patients with lower limb fractures.

## Author contributions

**Conceptualization:** Sung Il Wang, Yeon-Hee Han.

**Data curation:** Hwan-Jeong Jeong, Seok Tae Lim.

**Formal analysis:** Hwan-Jeong Jeong, Yeon-Hee Han.

**Methodology:** Sung Il Wang, Yeon-Hee Han.

**Project administration:** Hwan-Jeong Jeong.

**Resources:** Seok Tae Lim.

**Software:** Seok Tae Lim.

**Supervision:** Yeon-Hee Han.

**Validation:** Hwan-Jeong Jeong, Seok Tae Lim.

**Visualization:** Yeon-Hee Han.

**Writing – original draft:** Sung Il Wang, Yeon-Hee Han.

**Writing – review & editing:** Hwan-Jeong Jeong, Seok Tae Lim.
